# The NHS Long Term Workforce Plan: an ambitious leap or a misstep?

**DOI:** 10.1177/01410768231198373

**Published:** 2023-09-12

**Authors:** Tomas Ferreira, Alexander M Collins

**Affiliations:** 1 Department of Clinical Neurosciences, School of Clinical Medicine, University of Cambridge, Cambridge, CB2 0PY, UK; 2 School of Public Health, Faculty of Medicine, 4615 Imperial College London, London, W2 1PG, UK

The NHS Long Term Workforce Plan (LTWP), published in July 2023, presents an extensive framework designed to confront issues currently facing the National Health Service (NHS) and improve patient care in the United Kingdom (UK).^1^ With escalating demands on health services, for example, due to an ageing population and projected staffing shortfalls, urgent action is needed. This commentary seeks to critically evaluate certain aspects of the plan, providing an insight into its potential implications for the NHS and its workforce.

The plan's strategic direction encompasses short-, medium- and long-term goals in three domains: ‘train', ‘retain' and ‘reform'. Under ‘train', the government plans to double the number of medical school places by 2031/32, addressing geographical inequity by focusing increases in areas with pronounced shortages. Additionally, the plan proposes to introduce medical degree apprenticeships, with pilots commencing in 2024/25, pledging that by 2031/32, 2000 medical students will undergo training via this route. The ‘reform’ element includes strategies to shorten existing five- or six-year undergraduate medical degrees to four years and to increase numbers of physician associates (PAs) and anaesthesia associates (AAs), while also expanding their scope of practice.

## Recruitment versus retention

In 2021, approximately 10,000 doctors relinquished their licence to practise, representing a loss of nearly one-tenth of the total doctor workforce in the NHS.^2,3^ In addition to this, recent surveys report that almost half of doctors and consultants are actively planning to leave the NHS.^4,5^ Although plans to double medical school places attempt to counteract this deficit, this strategy could be likened to ‘filling a leaking bucket' if issues instigating the current exodus are not adequately addressed. Moreover, there is a risk that newly recruited graduates could be similarly inclined to leave unless systemic problems are rectified. The NHS's future success hinges not just on incoming fresh talent but also on the retention of its existing workforce and their valuable experience. Continual staff turnover could disrupt care continuity and potentially dilute the collective expertise within the NHS. There are also fiscal considerations associated with constant recruitment and training of new staff who leave soon thereafter, which could further strain an already beleaguered system.

The plan has proposed several strategies, including two likely contentious suggestions: a four-year fast-track medical degree and a medical apprenticeship programme for school leavers. However, important concerns have been raised regarding the quality of medical training and international recognition of UK qualifications. Establishing alternative training pathways could create a ‘two-tier' system of doctors, separating those who have undergone traditional medical training from those entering the profession via proposed undergraduate four-year or apprenticeship programmes. The concern is not only about internal perceptions within the NHS, where these doctors might be regarded as less experienced or less competent, but also about how UK-trained doctors are perceived internationally. Graduates of fast-track medical schools or apprenticeship programmes may find themselves ‘bound' to practise within the UK, as these qualifications might not be recognised abroad. This restriction could limit their career opportunities and mobility, potentially leading to increased dissatisfaction and fuelling further attrition.

Practical challenges in implementing the plan, such as increased training facilities or capacities to accommodate the increased number of medical students, could impact the quality of medical education. With fewer doctors available to train double the number of medical students, who assumes this enlarging responsibility? The anticipated pressure on the existing workforce could erode the quality of training and, subsequently, the quality of healthcare services. These requirements must be fulfilled to avoid overcrowded and inefficient working conditions, which could further exacerbate the existing stress on healthcare professionals.

In the existing system, obtaining a medical degree signifies the commencement of the journey towards specialisation. Upon graduation, individuals must complete a two-year foundation programme before they become eligible to apply for specialty training. Such an increase in the number of medical graduates could precipitate a highly competitive environment for scarce specialty training posts, potentially exacerbating existing competition ratios witnessed over the previous decade. Without corresponding increases in training posts, the situation could prove unsustainable, culminating in a large subset of non-specialised doctors. These highly trained individuals, initially aspiring to broaden their expertise through specialisation, could find themselves in a state of career stagnation, incapable of advancing. This may result in job dissatisfaction, paradoxically adding to existing challenges with staff retention.

## Professional scope and role of non-doctors

The LTWP outlines strategies to increase not only the number of PAs and AAs but also to expand their scope of practice. This brings into focus the concept of ‘scope creep', a gradual expansion of roles and tasks that professionals undertake, often extending beyond their traditional remit. By allowing non-doctors to take on tasks traditionally performed by doctors, it could liberate doctors to focus on more complex medical cases, increasing service capacity and providing greater flexibility, potentially enhancing overall system efficiency. However, if doctors are freed from such tasks only to focus on complex and demanding cases, the burden of care could become even more taxing. This redistribution of tasks may contribute to an increase in doctor burnout, which already presents an issue in the profession.

Further complicating the issue is the broader role of non-doctors. A critical consideration is patient safety and quality of care. PAs, for instance, typically undergo two years of medical education following completion of a bachelor’s degree, compared to a doctor's training that spans five or six years, followed by additional years of specialisation. Despite this difference in training, starting salaries for PAs significantly exceed that for new doctors, thereby leading to a disparity in the compensation structure that may not reflect the level of education or expertise. There is inherent risk in conferring complex medical tasks to relatively less-trained personnel, which might compromise patient safety or quality of care. The professional boundaries between doctors and these expanded roles may blur, potentially leading to confusion or tension among medical professionals, thereby influencing team dynamics. Further, there is a need to address the potential for confusion among patients about who is providing their care and their respective qualifications.

Moreover, when non-doctors undertake tasks traditionally reserved for doctors, establishing where responsibility lies in instances where treatment outcomes are suboptimal or result in harm is vital for patient safety, professional standards and legal clarity. This ambiguity could create further tension within the workforce and healthcare system. Mitigating these risks will require thoughtful strategies, including robust training and supervision arrangements for allied healthcare professionals (AHPs). However, it is essential to consider who will undertake this additional supervisory role, given doctors’ already substantial – and increasing – workloads. Adding further responsibility could compound doctors’ stress and potentially undermine the system's resilience.

## Conclusion and recommendations

In reflecting upon this proposal for the NHS, it becomes evident that a balance between the recruitment of new professionals and the retention of current staff is critical for the sustainability of the healthcare system. The strategy outlined indeed offers potential benefits, including increased service capacity and the fostering of diverse skill sets within the healthcare workforce. However, this plan also poses significant challenges, including ‘scope creep’, questions regarding international recognition of UK qualifications, infrastructure demands and the practical implications of considerable workforce expansion.

One prominent concern is the evolving scope of practice of AHPs. It is essential to establish clear and firm boundaries for this professional group and to subject these to regular reviews, anchored in robust evidence. Furthermore, necessary safeguards must be instituted to protect training opportunities available to doctors and prevent deskilling.

Addressing retention is critical to break the revolving door of healthcare professionals within the NHS. To incentivise staff retention, remuneration packages must be evaluated and adjusted to remain competitive on the international stage. If salaries within the NHS fail to match those offered elsewhere, such as Ireland and Australia, the risk of further loss of professionals becomes a plausible reality. Moreover, investment in infrastructure, including office spaces and training facilities, is necessary. A conducive work environment, inclusive of sufficient rest and work spaces, is not just a requisite for efficient workflow but also a critical determinant of staff morale and job satisfaction. The perception of being valued and respected within the workplace is a significant driver for staff retention.

The proposed increases in medical schools’ student capacity align well with escalating healthcare demands nationally. However, the logistical implications demand thoughtful consideration. These include the need for sufficient staff to educate the additional students, adequate facilities for their training and an increased number of specialty training posts and consultant positions to prevent professional bottlenecks. The medical apprenticeship programme and fast-track medical degrees raise similar concerns, as well as whether these routes will be recognised internationally.

In conclusion, healthcare workforce planning is an intricate process that requires careful consideration and execution. The initiatives outlined by the government offer possible solutions to some of the key issues facing the NHS, but these must be approached cautiously, considering both implementation and possible consequences. The path towards a robust, sustainable and efficient NHS workforce requires ongoing assessment, adaptation and a readiness to adjust strategies in response to evolving circumstances.

## Declarations

### Guarantor

TF.

### Transparency declaration

TF, the lead author, affirms that the article is an honest, accurate and transparent account of the study being reported; that no important aspects of the study have been omitted; and that any discrepancies from the study have been explained.

### Provenance

Not commissioned; editorial review.
